# 

**Published:** 1803-05-01

**Authors:** 


### To CORRESPONDENTS.

We have received some very favourable accounts of the success of the *Lichen Islandicus*, in consumptive cases; but the instances where the patients would not submit to regimen, or persevere in the use of the remedy, are more numerous. It is no part of the English character to submit to regimen, or persevere in the use of remedies apparently simple. There are other great obstacles in the way of the cure of Phthisis. Its attacks occur in that period of life when caution and prudence are rare; in that temperament where beauty and vivacity predominate; and the disease itself is generally attended with inordinate hope and good spirits. All these are directly opposed to the care and precautions necessary to the prevention or cure of Consumption.

Our Correspondents will, doubtless, attend to the Hints of Dr. Beddoes (p. 460) in their Communications respecting the Influenza.

Papers are received from Dr. Woodforde, Messrs. Harrup, Ramsay, Weston, O'Berne, Baker, Goodwin, and Wagstaffe.; several of them on the Influenza.

The Analysis of Calculi is obliged to be postponed, on account of the difficulty of colouring the Plate accurately. It will be given in the next Number.

